# Prolonged Survival and Phenotypic Correction of *Akp2*^−/−^ Hypophosphatasia Mice by Lentiviral Gene Therapy

**DOI:** 10.1002/jbmr.201

**Published:** 2010-08-04

**Authors:** Seiko Yamamoto, Hideo Orimo, Tae Matsumoto, Osamu Iijima, Sonoko Narisawa, Takahide Maeda, José Luis Millán, Takashi Shimada

**Affiliations:** 1 Department of Biochemistry and Molecular Biology, Nippon Medical SchoolTokyo, Japan; 2 Department of Pediatric Dentistry, Nihon University Graduate School of Dentistry at MatsudoMatsudo, Japan; 3 Sanford-Burnham Medical Research InstituteLa Jolla, CA, USA

**Keywords:** Alkaline phosphatase, Lentiviral vector, Enzyme replacement, Epilepsy, Calcification

## Abstract

Hypophosphatasia (HPP) is an inherited systemic skeletal disease caused by mutations in the gene encoding the tissue-nonspecific alkaline phosphatase (*TNALP*) isozyme. The clinical severity of HPP varies widely, with symptoms including rickets and osteomalacia. *TNALP* knockout (*Akp2*^−*/*−^) mice phenotypically mimic the severe infantile form of HPP; that is, *TNALP*-deficient mice are born with a normal appearance but die by 20 days of age owing to growth failure, hypomineralization, and epileptic seizures. In this study, a lentiviral vector expressing a bone-targeted form of *TNALP* was injected into the jugular vein of newborn *Akp2*^−*/*−^ mice. We found that alkaline phosphatase activity in the plasma of treated *Akp2*^−*/*−^ mice increased and remained at high levels throughout the life of the animals. The treated *Akp2*^−*/*−^ mice survived for more than 10 months and demonstrated normal physical activity and a healthy appearance. Epileptic seizures were completely inhibited in the treated *Akp2*^−*/*−^ mice, and X-ray examination of the skeleton showed that mineralization was significantly improved by the gene therapy. These results show that severe infantile HPP in *TNALP* knockout mice can be treated with a single injection of lentiviral vector during the neonatal period. © 2011 American Society for Bone and Mineral Research.

## Introduction

Hypophosphatasia (HPP) is an inherited skeletal disease caused by mutations in the gene encoding the tissue-nonspecific alkaline phosphatase (*TNALP*) isozyme.(1) The symptoms of HPP include hypomineralization that causes rickets in infants and children and osteomalacia in adults.(2,3) The clinical severity of HPP varies widely from a lethal perinatal form to mild odontohypophosphatasia that manifests only dental abnormalities.(4) In the infantile form, postnatal development appears to proceed normally before the onset of failure to thrive and the associated development of rickets before 6 months of age. Severe infantile HPP is often fatal.(3)

TNALP is an ectoenzyme that is attached to the outer plasma membrane via a glycosylphosphatidylinositol (GPI) anchor.(5,6) Absence of TNALP activity results in extracellular accumulation of natural substrates such as inorganic pyrophosphate (PP_*i*_ ),(7,8) pyridoxal 5'-phosphate (PLP),(9,10) and phosphoethanolamine (PEA).(11) Since high concentrations of PP_*i*_ result in a strong inhibition of hydroxylapatite crystal growth, normal mineralization of the systemic bones and teeth is impaired in HPP patients.(8,12) Pyridoxine-responsive seizures are also observed in some severe cases. Enzyme-replacement therapy using various types of alkaline phosphatase(13–17) and cell therapy using bone marrow cells(18,19) and mesenchymal cells(20) have been reported with no or very limited clinical benefit.

*TNALP* knockout mice have been established in two independent laboratories.(21,22) These mice are born with a normal appearance but, owing to deficient degradation of PP_*i*_ and abnormal metabolism of PLP, develop rickets and die by 20 days of age as a result of severe skeletal hypomineralization and epileptic seizures and represent an appropriate model of the infantile form of HPP.(23,24) Recently, Millán and colleagues(25) treated *TNALP* knockout mice with a daily subcutaneous injection of a bone-targeted form of TNALP in which a bone-targeting deca-aspartate sequence was linked to the C-terminal end of soluble TNALP.(26,27) Based on those data, clinical trials of enzyme-replacement therapy with bone-targeted TNALP in patients with adult and infantile HPP have been initiated.(28) A limitation of enzyme-replacement therapy for HPP is the restricted half-life of the TNALP protein in patients' fluids and tissues, which necessitates repeated administration of large amounts of the enzyme for long-term correction.

In this study, we examined viral vector–mediated gene therapy of HPP. We found that a single injection of lentiviral vector expressing bone-targeted TNALP into neonatal HPP mice resulted in long-term high levels of ALP in the serum and long-term phenotypic correction in HPP mice. We conclude that gene therapy may prove to be an important option for the treatment of human HPP.

## Materials and Methods

### Plasmid construction

To create *TNALP-D10* cDNA coding for TNALP lacking the GPI anchor sequence and containing 10 repeated aspartic acid (Asp) residues at its C-terminus, polymerase chain reaction (PCR) was performed using primers *TNALP-D10-*f (5'-GAA TTC ACC CAC GTC GAT TGC ATC TCT CTG GGC TCC AG) and *TNALP-D10-*r (5'-GAA TTC TCA GTC GTC ATC ATC ATC ATC GTC GTC ATC GTC GTC GCC TGC GGA GCT GGC AGG AGC ACA GTG-3') with pcDNA3 *TNALP* cDNA plasmid as the template.(29) The PCR product then was digested with *Eco*RI and inserted into the pGEM T-easy vector (Promega Corporation, Madison, WI, USA). A second PCR was performed using primers *Eco*RI–*TNALP-*f (5'-TTT GAA TTC GCC ACC ATG ATT TCA CCA TTC TTA GTA C-3') and *TNALP-D10*-*Not*I-r (5'-TTT GCG GCC GCT CAG TCG TCA TCA TCA TCA TCG). The orientation of each sequence then was confirmed.

**Fig. 1:**
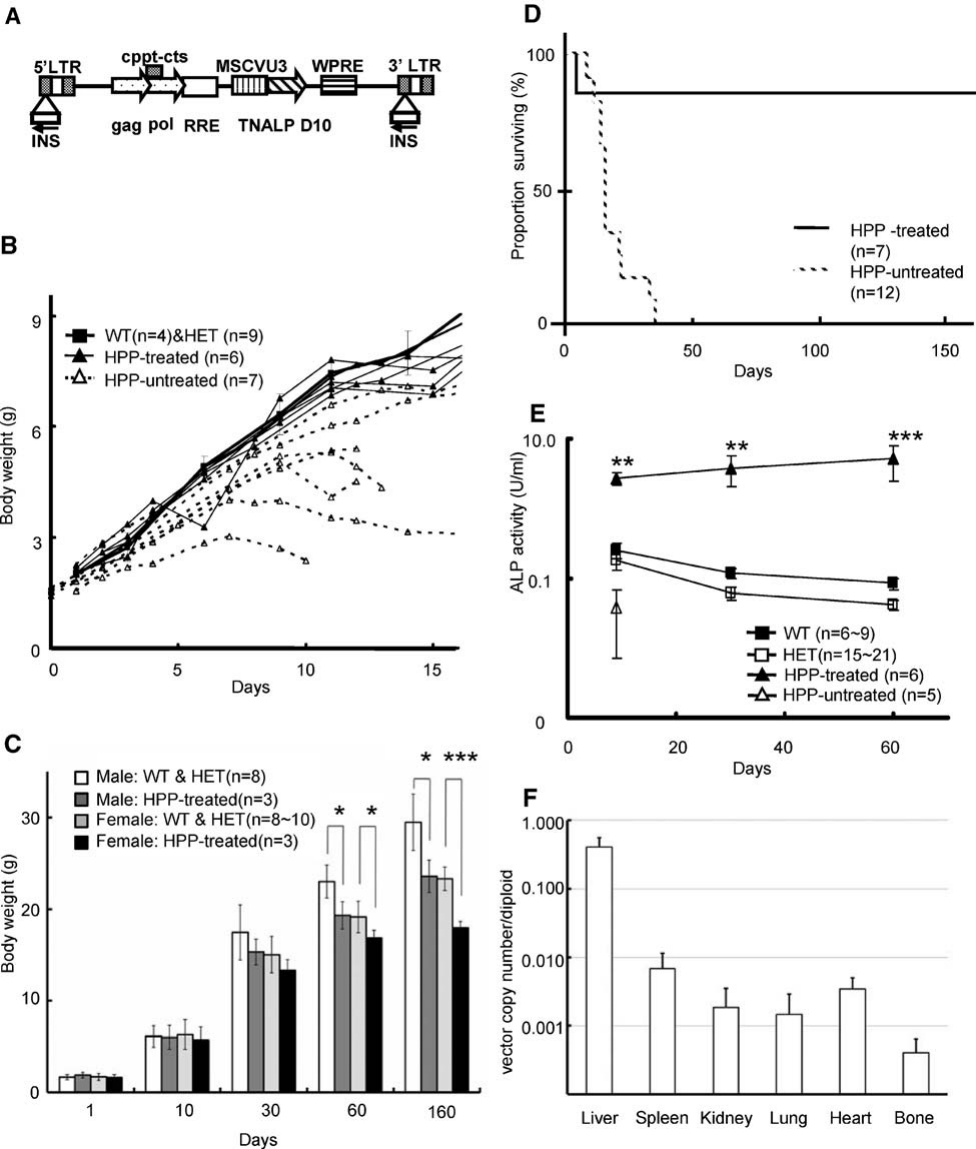
Lentiviral-mediated gene therapy of *Akp2*^−*/*−^ hypophosphatasic (HPP) mice. (*A*) Schematic diagram of HIV-TNALP-D10 lentiviral vector. LTR = long terminal repeat; MSCVU3 = U3 region of the LTR promoter of murine stem cell virus; WPRE = woodchuck hepatitis virus posttranscriptional regulatory element; INS = chicken β-globin hypersensitivity site 4 insulator; cppt-cts = central polypurine tract–central termination sequence; RRE = reverse responsive element. (*B*) Growth curves of untreated HPP mice (*n* = 7), treated HPP mice (*n* = 6), and WT (*n* = 4) and HET (*n* = 9) mice. The body weights of untreated HPP mice were recorded until spontaneous death. The weights of WT and HET (total *n* = 13) mice are presented as the average ± SD. (*C*) The comparison of average body weights of treated HPP mice (male, *n* = 3; female, *n* = 3) and WT/HET littermates (male, *n* = 8; female, *n* = 8 to 10). ^*^p < .05; ^***^p < .001. (*D*) The survival curves of treated (*n* = 7) and untreated (*n* = 12) HPP mice. (*E*) Concentration of plasma ALP in the treated (*n* = 6) and untreated (*n* = 5) HPP mice and HET (*n* = 15 to 21) and WT (*n* = 6-9) controls. ^**^p < .01 versus the WT group; ^***^p < .001 versus the WT group. (*F*) Distribution of lentiviral vector. The copy numbers of the vector genome in the organs was determined by qPCR with HIV-TNALP-D10 injected WT mice. Data are presented as mean ± SEM (*n* = 4).

The pHIV-TNALP-D10 plasmid was constructed by insertion of the *Eco*RI and *Not*I fragments containing the cDNA for *TNALP-D10* into pC1(–)3UTR-del, which is a newly constructed SJ1-based HIV-1 vector containing 0.25-kb insulators in the U3 and the murine stem cell virus (MSCV) long terminal repeat (LTR) as an internal promoter (Fig. 1*A*).(30)

### Lentiviral vector preparation

Lentiviral vector was prepared by transient transfection in 293T cells, as described previously.(30) Vector preparation treated with Benzonase (50 µL/mL) for 1 hour at room temperature was filtrated by 0.45-µm membrane after adjustment of the pH to 8.0 with 1 N NaOH. Vector was concentrated using Acrodisc Units with Mustang Q Membranes (PALL Corporation, Ann Arbor, MI, USA).(31,32) The eluted solution containing lentiviral vector was ultracentrifuged with a 20% (w/v) sucrose underlay for purification, and the infectious vector particle (titer) was determined in HeLa cells. The titer was expressed as transducing units per milliliter (TU/mL).

### Animal procedures and experiments

All animal experiments were preapproved by the Nippon Medical School Animal Ethics Committee. Wild-type (WT) *Akp2*^*+/−*^ heterozygous (HET) and *Akp2*^−*/*−^ knockout (HPP) mice were obtained by mating *Akp2*^*+/−*^ heterozygous mice with mice of a mixed 129J × C57Bl/6J genetic background.(22) Lentiviral vector (5.0 × 10^7^ TU/100 µL in PBS) was injected into the jugular vein of neonatal mice on days 1 through 3. Breeding HET pairs were fed modified Laboratory Rodent Diet 5001 (Purina Mills, St Louis, MO, USA)(35) composed of CMF laboratory feed (Oriental Yeast Co., Ltd., Tokyo, Japan) supplemented with 325 ppm pyridoxine/10 kg of feed.

### ALP activity

Blood samples were collected from the tail vein or the orbital sinus. The level of ALP in the plasma was quantified using a colorimetric assay for ALP activity, as described previously.(36) ALP activity was determined using 10 mM *p*-nitrophenyl phosphate (Sigma-Aldrich, Steinheim, UK) as the substrate in 100 mM 2-amino-2-methyl-1,3-propanediol-HCl buffer containing 5 mM MgCl_2_ (pH 10.0) at 37°C. ALP enzyme activity was described in units (U) defined as the amount of enzyme needed to catalyze production of 1 µmol of *p*-nitrophenol formed per minute. ALP activity in plasma was calculated as units per milliliter (U/mL).

### Biodistribution of lentiviral vector

Mice were deepley anesthesized and perfused with 15 mL of PBS containing 150 U of heparin and 15 mL of PBS. The liver, spleen, kidney, lung, heart, and bone (femur) were harvested, and homogenates were made using the Percellys-24 bead-beating homogenizer according to the company's protocol (Bertin Technologies, Paris, France). Genomic DNA was extracted from tissue homogenates using the Gentra Puregene Kit (Qiagen Siences, Germantown, MD, USA) and was subjected to real-time PCR to estimate the distribution. The primer/probe sets FPLV2 (modified at one base to 5'-ACT TGA AAG CGA AAG GGA AAC-3' owing to a difference in the HIV-1 strain), RPLV2 (5'-CAC CCA TCT CTC TCC TTC TAG CC-3'), and LV2 (5'-AGC TCT CTC GAC GCA GGA CTC GGC -3') were used to detect the lentiviral vector provirus, as described previously.(33) TaqMan ribosomal RNA control reagents (Applied Biosystems, Branchburg, NJ, USA) were used to quantify the amount of genomic DNA. To estimate vector distribution, genomic DNA extracted from the bone marrow cells of BL/6 wild-type mice or genomic DNA spiked with plasmid DNA was used as a standard, and average copy number per diploid were determined.(34)

### X-ray analysis

Digital microradiography images were obtained using a µFX-1000 (Fujifilm, Tokyo, Japan) and imaged with FLA-7000 (Fujifilm). The X-ray energy levels were 25 kV and 100 µA, and an exposure time of 90 seconds was used for 15-day-old mice, and 15 seconds was used for adult mice. We examined a minimum of three X-ray pictures from each group at given ages.

### ALP activity staining

Bone samples were fixed in neutral buffered formalin for 24 hours at 4°C. Knuckle samples then were decalcified in 10% EDTA solution with rotation for 2 to 3 days at 4°C. For the azo-dye method, knuckles were embedded in optimal-cutting-temperature (OCT) compound (Tissue-Tek, SAKUSA Finetechnical, Tokyo, Japan) and sectioned using a Leica CM1950 cryostat. Thin sections (4 µm thick) were air-dried for 10 minutes, washed in PBS, and transferred to a solution of 50 mM MgCl_2_ in 0.05 M Tris–maleic acid buffer (pH 7.4) for 30 minutes for the reactivation of ALP.(37) The sections then were incubated in a freshly prepared mixture of Naphthol AS-MX phosphate disodium salt (Sigma-Aldrich) and Fast Blue BB Salt (Sigma-Aldrich) as described previously.(38) Methyl green served as the counterstain.

### Statistical analysis

Data are expressed as mean ± SD. Differences between two groups were tested for statistical significance using Student's *t* test. *p* values < .05 were considered statistically significant. Kaplan-Meier curves were produced and analyzed using SPSS for Windows, Version 14.0J (SPSS Japan, Tokyo, Japan).

## Results

### Growth and survival of *Akp2*^−*/*−^ mice

The growth of the *Akp2*^*+/−*^ HET mice appeared indistinguishable from that of the WT mice. The *Akp2*^−*/*−^ HPP mice were born with a normal appearance and weight. However, HPP mice showed apparent growth failure and became progressively exhausted (Fig. 1*B*). Most of the HPP mice also developed spontaneous seizures with various clinical presentations, including tonic-clonic convulsions and abnormal running and vocalization. The mice usually died 1 to 2 days after the epileptic seizures began. The average life span of the HPP mice was 12.0 ± 4.4 days (*n* = 13). Pyridoxine supplementation of the food for the nursing mother delayed the onset of the epileptic attacks in the neonates and extended their survival to postnatal day 18.1 ± 7.6 (*n* = 15).

Lentiviral vector containing bone-targeted human *TNALP* cDNA (HIV-TNALP-D10) was injected into the jugular vein of the neonatal HPP mice on days 1 through 3 (*n* = 6). The weight and growth rates of the treated HPP mice were improved compared with the untreated HPP mice and were indistinguishable from those of their WT and HET littermates (*n* = 13; Fig. 1*B*). The long-term follow-up was done for 7 treated mice. Compared with untreated mice (*n* = 12), the life spans of treated mice (*n* = 7) were significantly extended up to at least 160 days of age, except that one treated animal died on day 6 from unknown causes (Fig. 1*D*). In the long survivors (*n* = 6), 3 were euthanized on day 160 for X-ray analysis, whereas the remaining 3 animals survived for more than 400 days with normal appearance and physical activity. Seizures were not observed in the treated mice throughout the experimental period. The average body weights of treated HPP (*n* = 6) and WT/HET (*n* = 13 to 18) were compared on days 1, 10, 30, 60, and 160 (Fig. 1*C*). The body weights differed between male and female mice after 60 days. In either gender, the slight but significant growth retardation was detected in treated HPP mice on days 60 and 160.

### Lentivirus-mediated expression of ALP

At 10 to 12 days after birth, ALP activity in the plasma of WT and HET mice was 0.25 ± 0.07 U/mL (*n* = 9) and 0.16 ± 0.05 U/mL (*n* = 21), respectively, whereas that of the HPP mice was less than 0.1 U/mL (*n* = 5; Fig. 1*E*). A single injection of HIV-TNALP-D10 into the neonatal HPP mice (*n* = 6) on days 1 through 3 resulted in extremely high levels of plasma ALP (2.67 ± 0.56 U/mL). The plasma ALP activity in the WT and HET mice decreased slowly with aging, whereas the lentivirus-mediated expression of ALP remained stable, and the high levels of ALP activity persisted for at least 6 months. At 60 days of age, the average ALP activity in the treated HPP mice was 73-fold higher than that of the WT mice (5.14 ± 2.66 versus 0.07 ± 0.02).

Biodistribution of lentiviral vector was determined using quantitative PCR (qPCR) on genomic DNA isolated from the injected WT littermate mice 14 days after injection (Fig. 1*F*). The highest copy number of integrated vector was detected in liver samples (0.40 copy/diploid). Low levels of lentiviral integration also were observed in the spleen and the heart. Transduction of the bone tissue, including bone marrow cells, was very low (<0.001 copy/diploid).

### Radiographic analysis

**Fig. 2:**
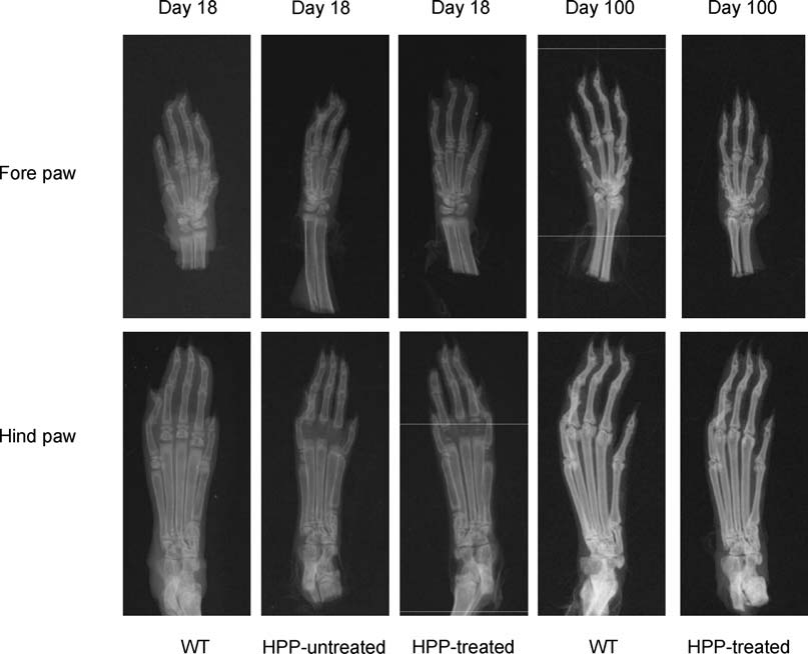
X-ray images of the feet. Secondary ossification centers in the hind paws were absent in untreated HPP mice but were detectable in the treated mice at 18 days after birth. No differences in skeletal mineralization were observed between treated long survivors and WT mice at 100 days of age.

Since radiographic changes in the HPP mice were not apparent during the first 8 days of life, we examined X-ray images of the feet and legs of mice at approximately 20 days after birth. The severity of the mineralization defects in the untreated HPP mice was found to be highly variable. In the most severely affected cases, the metacarpal and digital bones were significantly shorter than those of the WT mice, and their epiphyses were not detected. In addition, some of the carpal bones were absent. We also observed the absence of secondary ossification centers in the feet (Fig. 2). The most severe phenotype was observed in approximately 10% of the *Akp2*^−*/*−^ homozygous neonates, and these mice usually died by 10 days of age. In the milder cases, the epiphyses and all the digital bones were significantly mineralized, even though the HPP mice were smaller than the HET and WT mice. Heterogeneous radiographic changes between these two extreme phenotypes were observed in the untreated HPP mice.

X-ray images of treated HPP mice showed that mineralization was accelerated following lentivirus-mediated expression of TNALP-D10. Secondary ossification centers were detected in the feet of all treated animals at 15 days of age, although the intensity of the mineralization was variable. Ossification of the carpal bones also was improved. All the untreated HPP mice died, with an average survival of 18.1 days. No differences in skeletal structure and mineralization were observed between the long survivors after treatment and the WT mice at 100 days of age. These results indicate that mineralization defects in HPP mice can be corrected efficiently by gene therapy.

### Histochemical examination of the bone

**Fig. 3:**
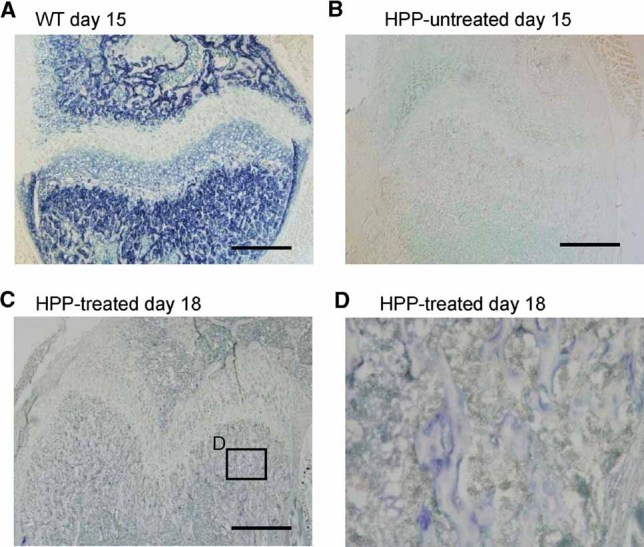
Histochemical staining of ALP activity in the tibias. ALP activity was detected in WT (*A*) but not HPP mice (*B*) at 15 days after birth. Following treatment with lentiviral vector, (*C*) ALP activity was detected on the surface of the endosteal bone at 18 days after birth. (*D*) Magnified image of the square in panel *C*. Bars = 1 mm.

The proximal tibias were analyzed histochemically for ALP activity using the azo-dye technique with methyl green counterstaining (Fig. 3). Strong ALP activity was detected in both the bone and hypertrophic cartilage zones of the WT mice (Fig. 3*A*), whereas no ALP signal was observed in the epiphysis of the HPP mice (Fig. 3*B*). After treatment with lentiviral vector, faint ALP staining was observed on the surface of the endosteal bone (Fig. 3*C, D*).

## Discussion

TNALP is an ectoenzyme that is known to be particularly abundant on the cell surfaces of osteoblasts and hypertrophic chondrocytes, including their shed matrix vesicles.(6,39) Since ALP functions on the exterior of the cell, enzyme replacement following repeated administration of soluble ALP has been hypothesized as a potential approach to treat ALP deficiencies. However, the outcomes of previous clinical trials of enzyme-replacement therapy have proven disappointing. Intravenous infusions of ALP-rich serum from patients with Paget disease(13,14) and purified soluble ALP from human liver(16) and placenta(17) have shown no significant clinical benefits in patients with HPP. Recently, Millán and colleagues(25) demonstrated that daily injections of high-dose bone-targeted TNALP significantly extended the lifespan and corrected the abnormal phenotypes of HPP mice, suggesting that HPP could be treated by enzyme replacement if sufficient amounts of TNALP were able to reach the sites of skeletal mineralization. Based on these data, new clinical trials involving enzyme-replacement therapy for HPP patients have been initiated.(28)

A general problem of enzyme-replacement therapy is the short half-life of the administered protein in patients. A pharmacokinetic study showed that the half-life of bone-targeted TNALP is 34 hours in the plasma of adult mice, but in bone tissue the half-life is extended to more than 300 hours.(25) Nevertheless, repeated administration of large amounts of the enzyme is required for long-term correction. In the initial clinical trials, HPP patients received subcutaneous injections of bone-targeted TNALP three times weekly.(28) The preparation of adequate amounts of clinical-grade purified enzyme is a limitation, and repeated injection is highly invasive and not optimal for small children. In this study we demonstrated that a single injection of lentiviral vector resulted in sustained expression of ALP and phenotypic correction in HPP neonatal mice. As such, viral vector–mediated enzyme replacement may prove to be more practical than classic enzyme replacement by repeated injection.

One of the concerns of gene therapy is the safety of the viral vector. We used an HIV-1-based lentiviral vector in this study.(30) Lentivirus-mediated gene transfer has proven to be effective for long-term expression of transgenes in nondividing cells. Although the pathogenicity of HIV-1 was almost negligible in the current modified version of lentiviral vector, insertional mutagenesis is still a major concern for all integrating vectors.(40) To minimize the possibility of protooncogene activation, our novel self-inactivating lentiviral vector contains the insulator element from the chicken β-globin locus.(30) So far, lymphoproliferative complications owing to insertional mutagenesis have been detected in ex vivo hematopoietic stem cell gene therapy only. For the treatment of HPP, lentiviral vector was injected directly into the circulation of neonatal mice. After this in vivo systemic gene therapy, the lentiviral sequence was detected in the liver, lung, and heart. The oncogenicity of the integrated lentiviral vector in these differentiated tissues requires further examination in a long-term follow-up study.

We also found that the epileptic seizures were completely inhibited and the lifespan was significantly extended in the treated HPP mice. Without treatment, HPP mice died by 20 days of age.(22,35) The major cause of death in the untreated HPP mice was apnea, most likely resulting from their severe epileptic convulsions.(21) Pyridoxine-responsive seizures in HPP patients and HPP model mice are thought to be caused by reduced levels of the inhibitory neurotransmitter γ-aminobutyric acid (GABA) in the brain.(21,35) PLP is an essential cofactor of glutamate decarboxylase, which is responsible for the synthesis of GABA.(41) Diminished hydrolysis of extracellular PLP in HPP causes decreased intracellular pyridoxal levels in cells. This results in a reduction in the rephosphorylation of pyridoxal to PLP, and thus biosynthesis of GABA within the brain cells is reduced.(10) The seizure phenotype can be rescued in part by administration of pyridoxal.(23) We demonstrated that epileptic seizures were efficiently inhibited by either systemic infusion of TNALP(25) or viral vector–mediated expression of TNALP, suggesting that the defective metabolism of PLP in the brain could be corrected by replacement of soluble TNALP.

Although epileptic seizures are observed in some severely affected patients, the major clinical complications in human HPP patients are directly related to defective skeletal mineralization.(3) Patients with severe infantile HPP usually die from respiratory failure caused by skeletal diseases in the chest, such as flail chest, rachitic deformity, and rib fractures.(3) Compared with severe infantile HPP in human patients, bone defects in our mouse model are relatively mild. The *Akp2*^−*/*−^ HPP mice were born with a normal appearance and bone mineral deposition. Hypomineralization becomes apparent after around 10 days of age, although the severity of mineralization defects varies widely.(22) Lentivirus-mediated expression of bone-targeted ALP efficiently prevents the progressive skeletal demineralization, as well as the lethal epilepsy.

Since enzyme replacement also was effective,(25) the major mechanism of successful gene therapy for HPP mice seems to be due to continuous supply of bone-targeted TNALP from the vector-infected liver to the circulation. Another possibility is that osteoblasts and chondrocytes may be directly transduced with lentiviral vector. However, since the copy number of integrated vector in the whole bone tissue was very low, the ALP staining in treated mice is mainly due to the circulating TNALP-D10 in the bone matrix. The contribution of in situ expression of TNALP in bone cells to bone mineralization is not likely to be significant.

A major physiologic role for TNALP has been shown to be the restriction of the extracellular pool of PP_*i*_, which is a strong inhibitor for mineralization.(24,39) Localization of TNALP to the skeleton should be important for the treatment of HPP. TNALP with a repetitive C-terminal extension of 10 Asp was shown to display high affinity for bone tissue both in vitro(26) and in vivo.(25) The use of the bone-targeted TNALP construct in a clinical setting is currently under investigation in clinical trials.(28)

The efficacy of gene therapy to correct hypomineralization was evaluated by radiographic examination. The major problem is that the severity of the mineralization defects in untreated infantile *Akp2*^−*/*−^ mice is highly variable. In addition, X-rays of infantile mice could be taken only after euthanization. The time course of mineralization in a single animal could not be examined under the condition used. Further studies using more reliable histomorphometric and micro–computed tomographic (µCT) techniques may be required to optimize the gene therapy protocol to rescue the skeletal phenotype.

In conclusion, we found that severe infantile HPP in *TNALP* knockout mice can be treated with a single injection of lentiviral vector during the neonatal period. Lentiviral-mediated gene therapy may prove to be an important option in the treatment of human hypophosphatasia.
